# Psychological correlates of quality of life in individuals with Vitiligo

**DOI:** 10.1192/j.eurpsy.2026.11043

**Published:** 2026-07-31

**Authors:** N. Manou, A. Papadopoulou, P.-D. Stavrou, E. Vousoura, V. Efstathiou

**Affiliations:** 1 Department of Psychology, National and Kapodistrian University of Athens; 2 Second Department of Psychiatry, “Attikon” University General Hospital, National and Kapodistrian University of Athens, Athens, Greece

## Abstract

**Introduction:**

Vitiligo is a chronic autoimmune dermatological condition characterized by the loss of melanin, resulting in depigmented macules (white patches) on the skin. Psychological distress has been linked to vitiligo both as a triggering factor and as a consequence.

**Objectives:**

The aim of the present study was to examine the relationship between psychological factors - namely depression, anxiety, self-esteem, mindfulness - and quality of life in individuals with vitiligo. The study also sought to investigate the possible impact of demographic and clinical characteristics, as well as perceived disease severity, on psychological factors and on multiple dimensions of quality of life, including physical and mental health, social relationships, environment, and level of independence.

**Methods:**

A total of 121 adults with vitiligo (81 women and 40 men) completed the following instruments: the Hospital Anxiety and Depression Scale, the Rosenberg Self-Esteem Scale, the World Health Organization Quality of Life - BREF, the Mindful Attention Awareness Scale - 15, and two self-report questionnaires assessing demographic and clinical information.

**Results:**

Higher levels of perceived disease severity were associated with increased anxiety and depression, as well as lower quality of life. Women reported higher anxiety and lower satisfaction with health than men, indicating greater psychological burden among women with vitiligo. Notably, self-esteem and mindfulness contributed positively to both physical and mental health. In multivariable models, physical health was independently related to lower depression and higher self-esteem; mental health was independently related to lower anxiety/depression and higher self-esteem/mindfulness; social relationships were negatively associated with age and depression, and positively with mindfulness; and environment/level of independence were negatively associated with earlier age at onset and positively with self-esteem and mindfulness (all p < .05). Facial depigmentation showed no significant main effects on psychological variables or quality of life, and no significant interaction with gender.

**Conclusions:**

The findings highlight the significant role of psychological factors in the quality of life of individuals with vitiligo and emphasize the need for a holistic, multidisciplinary approach to vitiligo management. Psychoeducation for healthcare professionals may serve as a valuable strategy to increase awareness and responsiveness, and psychotherapeutic interventions could benefit from incorporating mindfulness-based techniques.

**Disclosure of Interest:**

None Declared

